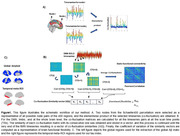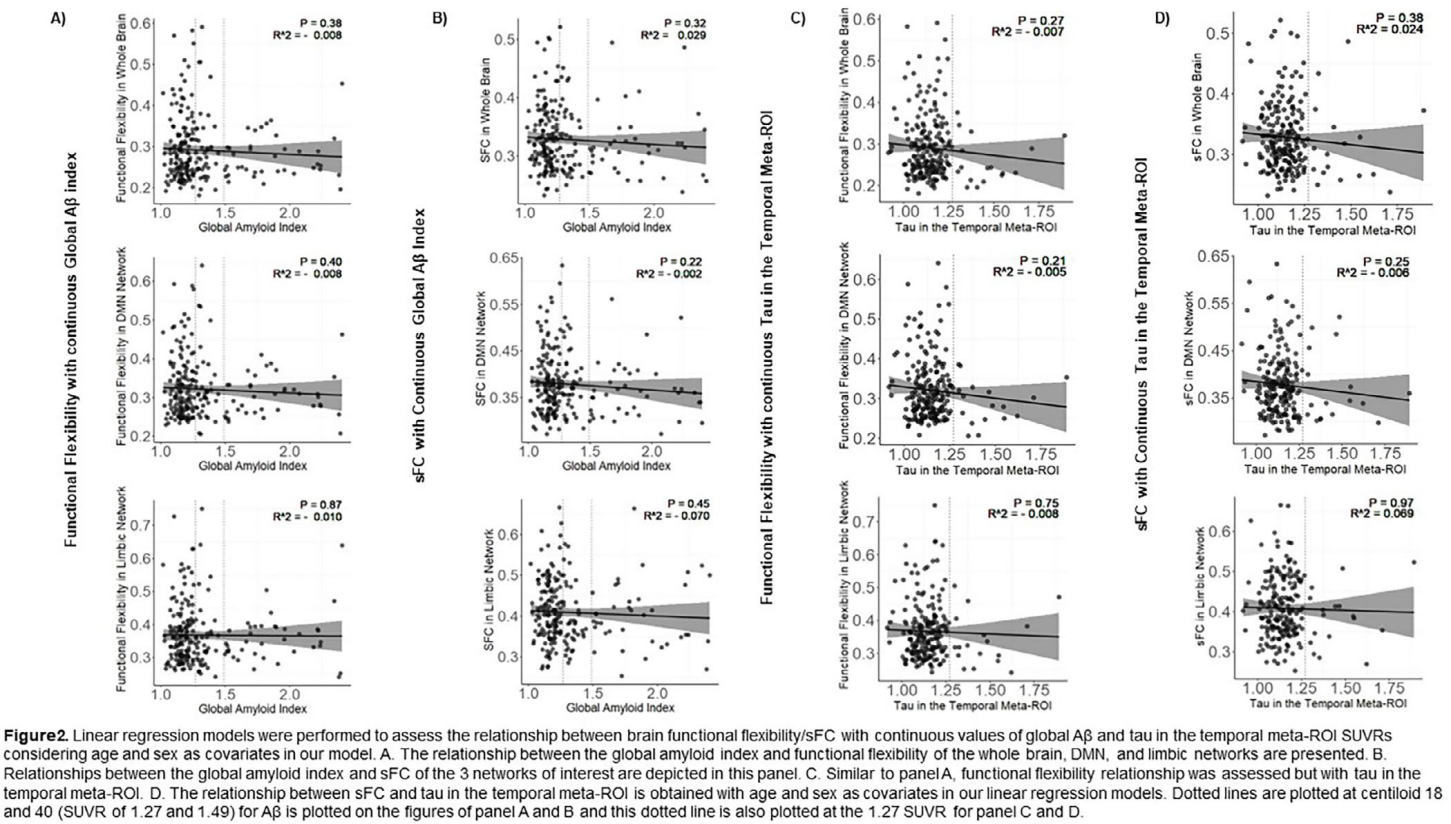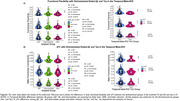# Brain Functional Flexibility and its Relationship to the Preclinical Stage of Alzheimer’s Disease

**DOI:** 10.1002/alz.091900

**Published:** 2025-01-09

**Authors:** Mohammadali Javanray, Frederic St‐Onge, Pierre Bellec, Bratislav Misic, Jean‐Paul Soucy, Sylvia Villeneuve, PEREVNT‐AD Research Group

**Affiliations:** ^1^ McGill University, Montreal, QC Canada; ^2^ Centre de Recherche de l'Institut Universitaire de Gériatrie de Montréal, Montréal, QC Canada; ^3^ Université de Montréal, Montréal, QC Canada; ^4^ Montreal Neurological Institute and Hospital (McGill University), Montreal, QC Canada; ^5^ Montreal Neurological Institute, McGill University, Montréal, QC Canada; ^6^ McConnell Brain Imaging Centre, Montreal Neurological Institute and Hospital, McGill University, Montreal, QC Canada; ^7^ The Douglas Research Center, Montreal, QC Canada; ^8^ Douglas Mental Health Research Centre, Montreal, QC Canada

## Abstract

**Background:**

Characterizing pathological and functional features of the preclinical stage of Alzheimer’s Disease (AD) is essential as Amyloid beta (Aβ) and tau, the pathological hallmarks of AD, start to accumulate years prior to the onset of clinical symptoms. Whether Aβ and/or tau are related to the brain’s ability to functionally reconfigure in time (functional flexibility) remains unclear despite its important role in behavior and cognition.

**Method:**

We included 233 cognitively unimpaired individuals with family history of AD from the PREVENT‐AD cohort who underwent both Positron Emission Tomography (PET) and functional Magnetic Resonance Imaging (fMRI). We computed the element‐wise product of the BOLD fMRI timeseries of the 400 Schaefer atlas nodes as co‐fluctuation matrices for the scan duration (all TRs). We then calculated the variabilities of the obtained co‐fluctuations at the whole brain and network level (DMN and limbic as early susceptible networks in AD) and used these measures of variability as a proxy of functional flexibility. We also computed the average of the obtained co‐fluctuation matrices, which mathematically corresponds to the Pearson correlation of the nodal timeseries pairs and used this as our marker of static functional connectivity (sFC). We then assessed the relationship between functional flexibility and sFC with the level of Aβ (global index) and tau (temporal meta‐ROIs), using those, both as continuous and dichotomized variables (Figure 1). Two thresholds were used for Aβ, one associated with low Aβ accumulation (centiloid of 18) and the other with significant Aβ burden (centiloid of 40).

**Result:**

No association was found between AD pathology and functional flexibility or sFC using AD pathology as continuous or dichotomized variables (Figure 2 and 3). However, the maximum range of functional flexibility values in individuals with significant Aβ burden and high tau was about half the one found in individuals with low or no pathology, a result that was particularly striking with tau.

**Conclusion:**

The absence of group difference suggests that functional flexibility cannot be used as a proxy of AD. While individuals with AD pathology have a low range of functional flexibility values, low values are also frequent in individuals with no pathology.